# A Dedifferentiation Strategy to Enhance the Osteogenic Potential of Dental Derived Stem Cells

**DOI:** 10.3389/fcell.2021.668558

**Published:** 2021-05-28

**Authors:** Francesco Paduano, Elisabetta Aiello, Paul Roy Cooper, Benedetta Marrelli, Irina Makeeva, Mohammad Islam, Gianrico Spagnuolo, Davide Maged, Danila De Vito, Marco Tatullo

**Affiliations:** ^1^Stem Cells and Medical Genetics Units, Biomedical Section, Tecnologica Research Institute and Marrelli Health, Crotone, Italy; ^2^Faculty of Dentistry, Sir John Walsh Research Institute, University of Otago, Dunedin, New Zealand; ^3^Department of Therapeutic Dentistry, I.M. Sechenov First Moscow State Medical University, Moscow, Russia; ^4^Department of Oral Surgery and Medicine, The Dental School, University of Dundee, Dundee, United Kingdom; ^5^Department of Neurosciences, Reproductive and Odontostomatological Sciences, University of Naples “Federico II,” Naples, Italy; ^6^EMS - Elite Medical Service Ltd.,, Cairo, Egypt; ^7^Department of Basic Medical Sciences, Neurosciences and Sense Organs, University of Bari “Aldo Moro,” Bari, Italy

**Keywords:** dental stem cells (DSCs), dental follicle progenitor stem cells (DFPCs), dental pulp stem cells (DPSCs), stem cell fate, dedifferentiation

## Abstract

Dental stem cells (DSCs) holds the ability to differentiate into numerous cell types. This property makes these cells particularly appropriate for therapeutic use in regenerative medicine. We report evidence that when DSCs undergo osteogenic differentiation, the osteoblast-like cells can be reverted back to a stem-like state and then further differentiated toward the osteogenic phenotype again, without gene manipulation. We have investigated two different MSCs types, both from dental tissues: dental follicle progenitor stem cells (DFPCs) and dental pulp stem cells (DPSCs). After osteogenic differentiation, both DFPCs and DPSCs can be reverted to a naïve stem cell-like status; importantly, dedifferentiated DSCs showed a greater potential to further differentiate toward the osteogenic phenotype. Our report aims to demonstrate for the first time that it is possible, under physiological conditions, to control the dedifferentiation of DSCs and that the rerouting of cell fate could potentially be used to enhance their osteogenic therapeutic potential. Significantly, this study first validates the use of dedifferentiated DSCs as an alternative source for bone tissue engineering.

## Introduction

Bone loss occurring after trauma or diseases is one of the main issues affecting the quality of life of many people worldwide (Paduano et al., 2017). Up to now, autologous bone transplantation has been considered the gold standard for the treatment for bone defects; however, it has several limitations, such as morbidity at the donor site and the limited availability of grafting material (Paduano et al., 2017). Therefore, new strategies are needed for the treatment and repair of bone defects.

Mesenchymal stem cells (MSCs) of dental origin, due to their multilineage differentiation ability, are considered a suitable cell source for clinical applications (Chatzivasileiou et al., 2013; Paduano et al., 2016; Ballini et al., 2017, 2018). However, there are several issues still to overcome before DSCs can be used in bone cell therapy. Dental stem cells (DSCs), like other MSC types, can exhibit a relatively low cell survival rate and differentiation potential *in vivo*, and this can significantly reduce their effectiveness in stem cell therapy, and thus their clinical usage (Liu et al., 2011; Rui et al., 2015). Consequently, it is of considerable interest to explore novel strategies to improve the regenerative commitment of DSCs. In this context, several researchers have demonstrated that the dedifferentiation process provides an interesting strategy for improving the ultimate differentiation potential of MSCs *in vivo* (Liu et al., 2011).

It has been hypothesised that fully differentiated mammalian cells are unable to undertake reverse cell differentiation, a process known as “dedifferentiation” (Zhang et al., 2010). However, recent evidence has shown that specific cells, such as epidermal cells, could be stimulated to dedifferentiate when exposed to specific stimuli, resulting in the reversion of cells to a less differentiated and more pluripotent state (Zhang et al., 2010). Indeed, it is also well known that somatic cells of human origin can be induced to dedifferentiate into induced pluripotent stem cells (iPSCs) by introducing the Yamanaka transcription factors *Klf4, c-Myc*, *Sox-2*, and *Oct4* (Pennock et al., 2015). However, until now, dedifferentiation of cells has been mainly obtained by nuclear reprogramming, which is a non-physiological process.

Recently, dedifferentiation has been considered for therapeutic application to enable the rerouting of cell fate by inducing the reversion of differentiated cells to a less differentiated state. This is subsequently characterised by an increased differentiation potential (Odelberg et al., 2000). In 2005, a study demonstrated that MSCs isolated from rat bone marrow, without gene manipulation, could be reprogrammed *in vitro* through osteogenic differentiation and dedifferentiation, resulting in cells having an increased capacity to form ectopic bone in nude mice (Rui et al., 2015). Following this dedifferentiation process, MSCs can be reverted to more primitive stem cells with improved osteogenic potential, cell survival, migratory capacity, colony-forming ability and increased expression of *Oct4*, *Sox2*, and *Nanog* (Rui et al., 2015). Furthermore, a more recent study has provided evidence that MSCs reprogrammed through neuronal differentiation and dedifferentiation are more therapeutically efficacious (Liu et al., 2011). These findings are clearly of significant interest as they potentially provide a physiological approach to overcome the limitations of iPSC, such as their genomic instability, tumorigenicity and immunogenicity (Lu and Zhao, 2013).

While some evidence shows that differentiated MSCs possess the ability to dedifferentiate into immature progenitors without genetic manipulation (Zhang et al., 2010; Sawa et al., 2019), no studies have shown whether DSCs have the ability to dedifferentiation into a stem cell-like state. Thus, this study investigated if DSCs can be dedifferentiated into a stem cell-like state without gene manipulation and if the dedifferentiation process can efficiently enhance the osteogenic capacity of DSCs *in vitro*.

This study aimed at evaluating the dedifferentiation strategy and its potential for enhancing the therapeutic efficacy of DSCs for bone-regenerative medicine applications.

## Results

### Osteogenic Dedifferentiated DFPCs

DFPCs used in this study express the typical MSC surface markers and possess the ability to form colony-forming units (CFU-F) and the multilineage differentiation potential (Supplementary Figures 1–3).

**FIGURE 1:**
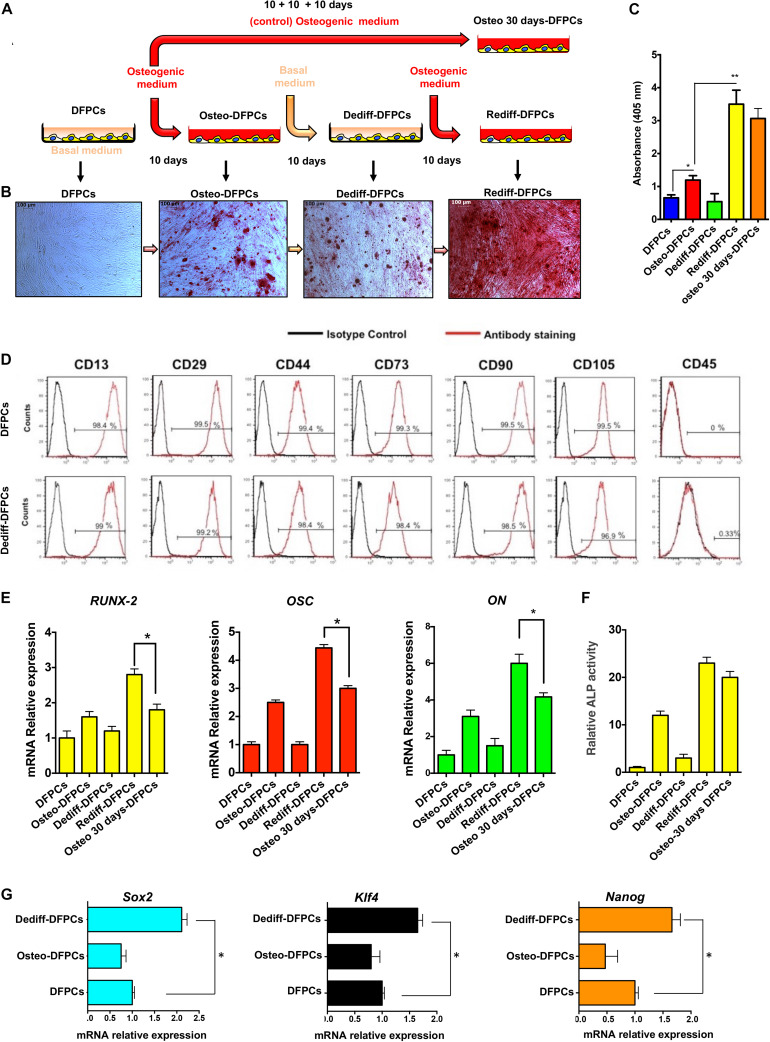
Osteogenic differentiation (Osteo-DFPCs), dedifferentiation (Dediff-DFPCs), and redifferentiation (Rediff-DFSCs) of DFPCs. **(A)** Schematic representation showing the technique for deriving Osteo-DFPCs, Dediff-DFPCs, and Rediff-DFPCs as described in the Materials and Methods. Briefly, undifferentiated DFPCs were stimulated for 10 days in osteogenic medium, rinsed in PBS, and then re-incubated for another 10 days with basal medium; redifferentiation was subsequently achieved using the osteogenic medium for another 10 days. **(B)** Alizarin Red stained images showing the presence of calcium deposition produced by DFPCs. The untreated DFPCs, Osteo-DFPCs, Dediff-DFPCs, and Rediff-DFPCs were incubated with (-MEM or osteogenic induced medium following the schematic provided in **(A)**, then DSCs were fixed and subjected to Alizarin Red staining. **(C)** Alizarin red quantification showing calcium deposition in Osteo-DFPCs, Dediff-DFPCs, and Rediff-DFPCs compared to DFPCs control. *P < 0.05 and **P < 0.01. DFPCs cultured in osteogenic medium for 30 days were used as a positive control for osteogenic differentiation (Osteo 30 days-DFPCs). **(D)** Cell surface markers of DFPCs and Dediff-DFPCs. **(E)** Relative mRNA expression levels of Runx2, OSC, and ON assayed by qRT-PCR in Osteo-DFPCs, Dediff-DFPCs, and Rediff-DFPCs compared to DFPCs control. HPRT mRNA levels were used for normalisation. The data are shown as mean ± SD (*n* = 3), *P < 0.05, compared to Osteo 30 days-DFPCs. **(F)** Relative ALP activity in Osteo-DFPCs, Dediff-DFPCs and Rediff-DFPCs compared to DFPCs control. **(G)** mRNA expression of the stemness-associated genes Sox2, Klf4, and Nanog in Dediff-DFPCs and Osteo-DFPCs compared with DFPCs. Data are shown as fold increase with respect to mRNA level expressed in undifferentiated DFPCs. *P < 0.05.

The methods used to drive the osteogenic differentiation, dedifferentiation and redifferentiation of DFPCs are schematically illustrated in Figure 1A. Briefly, undifferentiated DFPCs were induced in osteogenic medium for 10 days (Osteo-DFPCs), washed in PBS, and then re-incubated with the basal medium for a further 10 days (Dediff-DFPCs); redifferentiation was subsequently achieved using the osteogenic medium for another 10 days (Rediff-DFPCs, Figure 1A). To characterise the osteogenic commitment of DFPCs in each differentiation state, calcium deposits were assayed by Alizarin Red staining (Figure 1B). Withdrawal of osteogenic medium for 10 days reverted DFPC-derived osteogenic-like cells (Osteo-DFPCs) to cells with characteristic mesenchymal morphology and with reduced formation of mineralised nodules (Dediff-DFPCs). Intriguingly, these Dediff-DFPCs derived from differentiated osteogenic cultures (Osteo-DFPCs) could be re-induced into an osteogenic phenotype on re-exposure to osteogenic medium for 10 days (Rediff-DFPCs).

Importantly, results revealed that after 10 days of osteogenic induction medium, the presence of mineralisation was observed in both Rediff-DFPCs and Osteo-DFPCs. However, we detected a significantly higher Alizarin Red staining in Rediff-DFPCs than Osteo-DFPCs (Figure 1B). Also, spectrophotometric quantification of alizarin red staining indicated a higher mineralisation and calcium uptake in the Rediff-DFPCs than for the Osteo-DFPCs (Figure 1C). Importantly, Dediff-DFPCs retained their immunophenotype similar to that of undifferentiated DFPCs (Figure 1D).

Considering these interesting results, we subsequently compared osteogenic differentiation between Rediff-DFPCs and DFPCs cultured for 30 days in osteogenic medium (Osteo 30 days-DFPCs), the method widely used to differentiate MSCs toward an osteogenic lineage. Transcript levels of osteogenic-related genes were also assessed by qRT-PCR. As shown in Figure 1E, results demonstrated that the early inducer of osteogenic commitment runt-related transcription factor 2 (*Runx-2*), as well as the mature osteoblast marker osteocalcin (*OSC*) and bone-related protein osteonectin (*ON*), were significantly increased in Rediff-DFPCs compared with DFPCs cultured for 30 days in osteogenic medium (Osteo-30 days-DFPCs).

Taken together, these data demonstrated the enhanced expression of osteogenic-related transcripts in the Rediff-DFPCs derived from Dediff-DFPCs compared with Osteo 30 days-DFPCs. These data indicated that Dediff-DFPCs maintained osteogenic commitment, and therefore, a higher capacity to redifferentiate toward the osteogenic phenotype. In addition, as displayed in Figure 1F, Rediff-DFPCs also showed an increase of ALP activity compared with Osteo 30 days-DFPCs.

As *Sox2*, *KLf4*, and *Nanog* are key pluripotent genes necessary for reprogramming iPSCs and embryonic stem cell (ESC) pluripotency, we investigated their expression using qRT-PCR. Interestingly, results showed a significantly higher expression of *Sox2*, *KLf4*, and *Nanog* in Dediff-DFPCs than untreated DFPCs, suggesting a more primitive phenotype of dedifferentiated DFPCs (Dediff-DFPCs, Figure 1G). These results indicated that the stemness genes Sox2, Klf4, and Nanog play a crucial role in maintaining the self-renewal state in Dediff-DFPCs.

Interestingly, Dediff-DFPCs exhibited advantages in proliferation and clonogenicity over untreated DFPCs (Supplementary Figure 5).

### Dedifferentiated DPSCs Exhibit Enhanced Osteogenic Differentiation Capabilities

As DPSCs are the most widely studied and well-characterised DSC type [3,4], we investigated the osteogenic differentiation capacity of dedifferentiated DPSCs (Dediff-DPSCs). As observed previously for DFPCs, DPSCs used in this study also express the typical MSC surface markers and possess the ability to form colony-forming units (CFU-F) and the multilineage differentiation potential (Supplementary Figures 2–4).

Importantly, we compared the osteogenic potential of Rediff-DPSCs not only with that of undifferentiated DPSCs but also with that of DPSCs grown in osteogenic medium for 30 days (Osteo 30 days-DPSCs), the standard method used to differentiate DPSCs into osteogenic-like cells *in vitro* (Marrelli et al., 2013; Barry et al., 2016).

The approach used to stimulate osteogenic differentiation, dedifferentiation, redifferentiation and osteogenic differentiation for 30 days of DPSCs (Osteo 30 days-DPSCs) is schematically identical to Figure 1A. As observed previously for DFPCs, the Dediff-DPSCs derived from differentiated osteogenic cultures (Osteo-DPSCs) could also be re-induced into an osteogenic phenotype following re-exposure to osteogenic medium for 10 days (Rediff-DPSCs).

**FIGURE 2:**
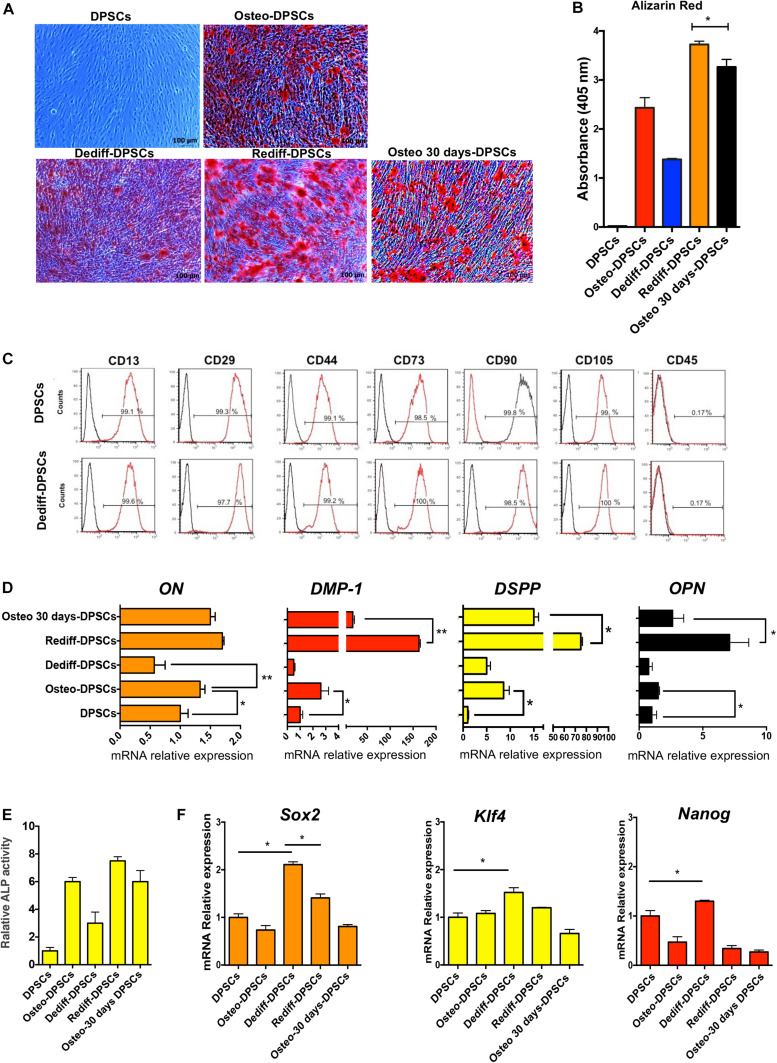
Osteogenic differentiation (Osteo-DPSCs), dedifferentiation (Dediff-DPSCs) redifferentiation (Rediff-DPSCs) of DPSCs and osteogenic differentiation for 30 days (Osteo 30 days-DPSCs) used as control. **(A)** Alizarin red stain images showing calcium deposition in DPSCs, Osteo-DPSCs, Dediff-DPSCs, Rediff-DPSCs, and Osteo 30 days-DPSCs. **(B)** Alizarin red quantification showing mineralised deposits in Osteo-DPSCs, Dediff-DPSCs, Rediff-DPSCs, and Osteo 30 days-DPSCs. **(C)** Cell surface markers of DPSCs and Dediff-DPSCs. **(D)** Relative expression levels of *ON, DMP-1, DSPP*, and *OPN* assayed by qRT-PCR in DPSCs, Osteo-DPSCs, Dediff-DPSCs, Rediff-DPSCs, and Osteo 30 days-DPSCs. mRNA *HPRT* levels were used as control. Results are shown as mean ± SD (*n* = 3), *P < 0.05; **P < 0.01. **(E)** Relative ALP activity in Osteo-DPSCs, Dediff-DPSCs, and Rediff-DPSCs compared to DPSCs control. **(F)** mRNA expression of the stemness-associated genes *Sox2, Klf4*, an*d Nanog* in Osteo-DPSCs, Dediff-DPSCs, Rediff-DPSCs, and Osteo 30 days-DPSCs with respect to DPSCs. *P < 0.05.

Importantly, results showed that after osteogenic medium induction, Alizarin Red positive stained calcium nodules formed in the Rediff-DFPCs were significantly greater than those observed in Osteo 30 days-DPSCs (Figure 2A). Spectrophotometric quantification of alizarin red staining demonstrated higher mineralisation in the Rediff-DPSCs than Osteo 30 days-DPSCs (Figure 2B). These data demonstrate that dedifferentiated DPSCs possesses greater potential for redifferentiation toward the osteogenic phenotype. Importantly, Dediff-DPSCs retained their immunophenotype similar to that of undifferentiated DPSCs (Figure 2C).

In addition, we observed that the mRNA expression levels of *osteonectin* (*ON*) and *osteopontin* (OPN), two markers of osteogenic differentiation, as well as transcript levels of two important markers of dental pulp cell differentiation, including *Dmp-1* and *Dspp* are were considerably increased during osteogenic differentiation (Osteo-DPSCs). Notably, the mRNA expression levels of all these genes decreased during the withdrawal of the osteogenic stimuli (Dediff-DPSCs, Figure 2D).

In addition, as displayed in Figure 2E, Rediff-DPSCs also showed an increase of ALP activity compared with Osteo 30 days-DPSCs.

The progression from osteogenic differentiated to undifferentiated cells is associated with a significant reduction in the expression of *ON, OPN, Dmp-1*, and *Dspp*. Notably, the mRNA levels of these markers were again upregulated in Rediff-DFPCs, cultures that were re-exposed to osteogenic medium.

As *Sox2*, *Klf4*, and *Nanog* are key pluripotent genes necessary for reprogramming iPSCs and ESC pluripotency (Pennock et al., 2015), we investigated their expression using a qRT-PCR assay. Interestingly, results showed a significantly higher expression of *Sox2, Klf4*, and *Nanog* in Dediff-DPSCs with respect to untreated DPSCs, demonstrating a more primitive phenotype of dedifferentiated DPSCs (Dediff-DPSCs, Figure 2F). The mRNA expression levels of these pluripotent genes were decreased during osteogenic differentiation (Osteo-DPSCs), whereas they were increased during the withdrawal of the osteogenic stimuli (Dediff-DPSCs). These results indicated that these stemness genes play a crucial role in maintaining the self-renewal state in Dediff-DPSCs. As observed previously for DFPCs, Dediff-DPSCs also exhibited advantages in proliferation and clonogenicity over untreated DPSCs (Supplementary Figure 5).

## Discussion

In our study, we analysed the osteogenic differentiation potential of redifferentiated DSCs (DFPCs and DPSCs) compared to osteogenic differentiated DSCs. Results showed that following osteogenic differentiation and dedifferentiation *in vitro*, both DSC types exhibited enhanced osteogenic differentiation potential. We also observed that the transcription factors *Sox2, Klf4*, and *Nanog*, key pluripotent genes, were upregulated after the dedifferentiation process of DSCs. Both dedifferentiated DSCs were characterised by an elevated mRNA expression level of *Sox2, Klf4*, and *Nanog*. It is well known that these transcription factors are essential for the maintenance of pluripotency, and therefore, we hypothesise that they could play a crucial role in the dedifferentiation process in these cells. Although it is well known that pluripotent genes such as *Sox2* and *Nanog* are critical for the maintenance of the pluripotency of embryonic stem (ES) cells and induced pluripotent stem cells (iPSC), it has been shown that these genes play a similar role also in adult stem cells (Rui et al., 2015). For example, the overexpression of *Nanog* and *Oct4* or Sox2 can improve the stemness and osteogenesis of human bone marrow MSCs (Go et al., 2008; Liu et al., 2009). Therefore, pluripotent genes are not only essential for self-renewal and pluripotency of ES cells and iPSCs, but also for MSCs such as DSCs.

Similar results were previously reported by Rui et al. (2015) who studied dedifferentiated bone marrow stem cells (BMSCs). In their work, they demonstrated that the mRNA expression levels of *Sox2* and *Nanog* were increased in dedifferentiated BMSCs compared with untreated cells. Our results, together with those described above, suggest that the improvement of MSC potency observed during the dedifferentiation process could be related to the increased expression of *Sox2* and *Nanog*. Indeed, the induced alteration of gene expression observed in dedifferentiated DSCs could confer an extended differentiation potential to these cells.

Importantly, here we also demonstrated for the first time that the modulation of culture conditions by altering the levels of osteogenic inducing factors could be used in human DSCs to induce the dedifferentiation process. Interestingly, this dedifferentiation strategy can increase the osteogenic differentiation capacity of DSCs compared with the standard method to differentiate DFPCs or DPSCs into osteogenic-like cells *in vitro*. Importantly, Rui et al. obtained similar results to ours and demonstrated that this differentiation strategy could be used to enhance the osteogenic therapeutic potential of BMSCs (Pennock et al., 2015; Rui et al., 2015).

Interestingly, it has been shown that epigenetic changes can regulate stem cell differentiation and dedifferentiation. Indeed, particular patterns of histone modifications and DNA methylation have an essential role in stimulating DPSC differentiation toward the osteogenic lineage (Paino et al., 2014). Two independent studies have demonstrated that histone deacetylase (HDAC) inhibition with valproic acid significantly improved mineralised matrix formation by improving the expression of bone glycoproteins involved in the formation of the mineralised matrix (Paino et al., 2014; Duncan et al., 2016). However, HDAC inhibitors could not easily be utilised *in vivo* to enhance the osteogenic differentiation potential of DPSCs. Thus, the development of an alternative strategy, such as the dedifferentiation process described here, which is able to reinforce the osteogenic differentiation potential of cells is needed.

While *in vitro* evidence indicates that dedifferentiated SCs provide a promising cell source for clinical applications, no clinical studies have yet been performed using this approach. Here, we have identified an alternative method to enhance the osteogenic potential of DSCs that could provide hypothetical clinical benefits with respect to the use of undifferentiated cells.

Naïve DSCs, like other MSC types, exhibit relatively low cell survival rates and differentiation potential *in vivo*. This finding can significantly reduce the effectiveness of stem cell therapies, and thus their clinical usage. To avoid this problem, we propose the use of cells having a higher osteogenic potential that can be relatively easily derived by using a physiological approach. The dedifferentiation process can be considered a physiological mechanism since it exists in various tissues and organs from animals, amphibians and plants (Yao and Wang, 2020). For example, cardiomyocytes and Schwann cells can dedifferentiate during cardiac regeneration and nerve injury, respectively (Yao and Wang, 2020). Thus, dedifferentiation is mechanistically associated with natural regeneration since it is part of the physiological response to injury in numerous organs (Merrell and Stanger, 2016).

Thus, due to their enhanced ability to differentiate toward the osteogenic commitment phenotype, instead of using naïve DSCs, we propose the use of dedifferentiated DSCs for bone therapeutic purposes.

The data generated in this study has the potential to be translated for the development of novel and more efficacious therapeutic approaches for human bone and dental tissue regeneration. Despite the scientific literature reporting several tissue engineering strategies involving MSCs (Paduano et al., 2016, 2017; Posa et al., 2016; Ballini et al., 2017, 2018; Marrelli et al., 2018) or biomimetic materials (Marrelli et al., 2013; Barry et al., 2016; Tatullo et al., 2018), biomedical researchers continue to face key difficulties which hinder translational approaches for bone regeneration, including issues relating to patient safety following extended cell manipulation (Marrelli et al., 2013; Cantore et al., 2016; Duncan et al., 2016; Marrazzo et al., 2016; Spagnuolo et al., 2020). Importantly, the approach described here requires a relatively simple and safe methodology that does not modify the biological characteristics of the cells.

A limitation of this study is the current lack of evaluation of the multi-differentiation potential of dedifferentiated DSCs. Currently, we have only focused on the evaluation of the osteogenic differentiation potential of dedifferentiated DSCs. We believe that the knowledge of DSC physiology in terms of dedifferentiation and redifferentiation could enhance the development of novel and more competent therapeutic strategies for human bone regeneration. The significance of this study is its exploration of the possibility to use dedifferentiated DSCs as an alternative stem cell source for bone tissue engineering. Importantly, our strategy requires the use of good manufacturing procedures that do not alter the biological features of these accessible cells and thus, it would also be ethically acceptable.

It is essential to underline that both types of DSCs studied were isolated using an enzymatic digestion strategy. Therefore, although we observed that the population of dedifferentiated DSCs possesses a phenotype distinct from their undifferentiated counterpart, we are aware that there is phenotypic heterogeneity in each DSC population. Consequently, the heterogeneity of the DSC population could make the generalisation of findings difficult, and therefore, future studies are necessary to generate a standardised protocol.

## Conclusion

Our results show for the first time that under physiological conditions, it is possible to induce the dedifferentiation of DSCs. Importantly, dedifferentiated DSCs exhibit an enhanced potential for osteogenic differentiation compared with their undifferentiated counterpart. Therefore, the dedifferentiation strategy could be potentially used to enhance the osteogenic therapeutic potential of DSCs. Furthermore, *in vivo* studies are necessary to evaluate the therapeutic efficacy of dedifferentiated DSCs *in vivo* and shed light on the molecular mechanisms involved in DSC dedifferentiation. In conclusion, we provide a novel approach in dental stem cell biology, in which the rerouting of DSCs fate could offer new therapeutic opportunities.

## Materials and Methods

### Isolation and Application of Dental Stem Cells

In this study, two types of human dental stem (DSCs) were studied and included: dental follicle progenitor stem cells (DFPCs) and dental pulp stem cells (DPSCs). Freshly isolated DFPCs and DPSCs were regarded as being at the undifferentiated stage.

DFPCs and DPSCs were isolated as previously described (Chatzivasileiou et al., 2013; Marrelli et al., 2015; Tatullo et al., 2015a,b; Paduano et al., 2016, 2017). DSCs were obtained from healthy volunteers, who provided informed consent at the *Calabrodental* Dental Clinic (Crotone, Italy). The study was performed under guidelines approved by the Ethical Committee at the Calabrodental Dental Clinic (Ethical agreement number CBD-001/TRI/2020). Briefly, impacted third molars were rinsed twice in PBS containing streptomycin (100 μg/mL) and penicillin (100 U/mL). Dental pulp tissues were separated from the crown and root, and the freshly extracted dental follicle was separated from the mineralised tooth. Collected dental pulp tissue and dental follicles were minced and immersed in a solution containing dispase (4 mg/mL) and type I collagenase (3 mg/mL) at 37°C for 60 min. Each cell suspension was filtered using Falcon strainers (70 μM) and incubated in basal media comprised of alpha-MEM culture medium containing 10% FBS (Invitrogen, Carlsbad, California, United States), streptomycin (100 mg/mL), glutamine (2 mM) and penicillin (100 U/mL). Both DSCs were incubated at 37°C with 5% CO_2_.

### Osteogenic Differentiation, Dedifferentiation and Re-differentiation Analysis of DSCs

Cells were plated into 100-mm cell culture dishes at a cell density of 3 × 10^5^ (DFPCs) and 4 × 10^5^ DPSCs until reaching a confluence of 60–70%. Then, to induce osteogenic differentiation, undifferentiated (freshly isolated) DFPCs and DPSCs were grown in osteogenic medium containing α-MEM (Sigma, St. Louis, MO, United States) supplemented with 50 μg/mL L-ascorbic acid, 5 mM β-Glycerophosphate, 10 nM dexamethasone and 20% FBS (Invitrogen, Carlsbad, CA, United States), for 10 days (Osteo-DFPCs and Osteo-DPSCs) according to previously described procedures (Marrazzo et al., 2016; Paduano et al., 2017). Subsequently, the osteogenic medium was replenished with basal media, and subsequently, cells were grown for a further 10 days (Dediff-DFPCs and Dediff-DPSCs). These cells were considered in a dedifferentiated stage (Dediff).

The basal medium was then again replaced with osteogenic medium for 10 days (Rediff-DFPCs and Rediff-DPSCs). Importantly, DFPCs and DPSCs cultured in osteogenic medium for 30 days were used as a positive control for osteogenic differentiation (Osteo 30 days-DPSCs). Mineralisation of DFPCs and DPSCs were observed on day 10 (Osteo-DFPCs and Osteo-DPSCs), at day 20 (Dediff-DFPCs and Dediff-DPSCs) and day 30 (Rediff-DFPCs, Rediff-DPSCs, Osteo 30 days-DPSCs. Since DFPCs exhibit a proliferative rate higher than DPSCs, instead of at 10 days, they can be induced toward the osteogenic lineage in the presence of osteogenic medium for 7 days.

Briefly, on the indicated day, cells were rinsed with PBS and fixed with paraformaldehyde 4% (Sigma, St. Louis, MO, United States) at room temperature for 20 min. A 5 mg/mL concentrated solution of Alizarin Red (Sigma, St. Louis, Missouri, United States) was added to both cell types for 30 min, and the quantification of Alizarin Red was obtained as we have previously described elsewhere [16]. Briefly, cell samples were cultivated in 10% acetic acid and boiled for 10 min. After centrifugation, samples were evaluated at a wavelength of 405 nm using a Multiskan Go Spectrophotometer (Thermo Fisher Scientific, Waltham, MA, United States) (Paduano et al., 2017).

### Gene Expression Analysis

RNA was isolated from DFPCs and DPSCs according to the *Purelink RNA mini kit* protocol (Applied Biosystem, United Kingdom), and the concentrations of extracted RNA were calculated using a spectrophotometer (Multiskan Go, Thermo Fisher Scientific, Waltham, Massachusetts, United States). Gene expression levels were measured by qRT-PCR, as previously described (Paino et al., 2014; Paduano et al., 2016, 2017; Tatullo et al., 2017, 2018). Briefly, RNA (200 ng) was reverse-transcribed according to the High Capacity cDNA Reverse Transcription Kit (Applied Biosystems, Foster City, California, United States). Subsequently, cDNA samples were amplified by qRT-PCR using specific primers supplemented with SYBR Green Master Mix. qRT-PCR reactions were performed using the following conditions: initial denaturation step at 95°C for 10 min, followed by 40 cycles of 10 s at 95°C and 1 min at 60°C. The specificity of PCR products was checked by melting curve analysis. After amplification, mRNA expression levels of the target genes were calculated by the *2-*ΔΔ*Ct* method. Hypoxanthine phosphoribosyltransferase (HRPT) was used as a control to normalise the levels of mRNA in all samples. See Table 1 for primer details.

**TABLE 1:** Primer details used for qRT-PCR analysis.

Gene symbol	Sequence (5′–3′)		NCBI accession number
*Runx-2^*a*^*	**Forward:**	ATGTGTGTTTGTTTCAGCAGCA	NM_001278478.2
	**Reverse:**	TCCCTAAAGTCACTCGGTATGTGTA	
*Klf4*^*b*^	**Forward:**	CCATCTTTCTCCACGTTCG	NM_004235.4
	**Reverse:**	AGTCGCTTCATGTGGGAG	
*Sox2*^*c*^	**Forward:**	GACTTCACATGTCCCAGCACTA	NM_003106.3
	**Reverse:**	CTCTTTTGCACCCCTCCCATT	
*Nanog*^*d*^	**Forward:**	ATTCAGGACAGCCCTGATTCTTC	NM_024865.3
	**Reverse:**	TTTTTGCGACACTCTTCTCTGC	
*HPRT*^*e*^	**Forward:**	TGACACTGGCAAAACAATGCA	NM_000194.2
	**Reverse:**	GGTCCTTTTCACCAGCAAGCT	
*ON*^*f*^	**Forward:**	TGCATGTGTCTTAGTCTTAGTCACC	NM_001309443.2
	**Reverse:**	GCTAACTTAGTGCTTACAGGAACCA	
*OSC*^*g*^	**Forward:**	TGAGAGCCCTCACACTCCTC	NM_199173.6
	**Reverse:**	ACCTTTGCTGGACTCTGCAC	
*DMP-1^*h*^*	**Forward:**	GTGAGTGAGTCCAGGGGAGATAA	NM_004407.3
	**Reverse:**	TTTTGAGTGGGAGAGTGTGTGC	
*DSPP*^*i*^	**Forward:**	CTGTTGGGAAGAGCCAAGATAAG	NM_014208.3
	**Reverse:**	CCAAGATCATTCCATGTTGTCCT	
*OPN*^*j*^	**Forward:**	CAGTTGTCCCCACAGTAGACAC	NM_001040058.1
	**Reverse:**	GTGATGTCCTCGTCTGTAGCATC	

**^*a*^Runx-2, RUNX family transcription factor; ^*b*^Klf4, kruppel-like factor 4; ^*c*^Sox2, SRY-related HMG-box 2; ^*d*^Nanog, homeobox transcription factor Nanog; ^*e*^HPRT, hypoxanthine phosphoribosyltransferase; ^*f*^ON, osteonectin; ^*g*^OSC, osteocalcin; ^*h*^DMP-1, dentin matrix acid phosphoprotein-1; ^*i*^DSPP, dentin sialophosphoprotein; ^*j*^OPN, osteopontin.**

### Alkaline Phosphatase (ALP) Assay

ALP activity was analysed using the alkaline phosphatase assay kit (ABCAM). Briefly, for each condition, cells were lysed and centrifuged. Supernatants were collected for the detection of ALP activity as described by the manufacturer. Absorbance was measured at 405 nm, and values were normalised to the total protein per volume of lysate.

### Statistical Analysis

Data are shown as mean ± SD from three independent experiments in triplicate. Results were analysed by GraphPad Prism software, and values were indicated statistically significant when ^∗^*P* < 0.05 and ^∗∗^*P* < 0.01.

## Data Availability Statement

The authors acknowledge that the data presented in this study must be deposited and made publicly available in an acceptable repository, prior to publication. Frontiers cannot accept a manuscript that does not adhere to our open data policies.

## Ethics Statement

The studies involving human participants were reviewed and approved by the Committee at the Calabrodental Dental Clinic (Ethical agreement number CBD-001/TRI/2020. The patients/participants provided their written informed consent to participate in this study.

## Author Contributions

FP and MT conceived the study. FP, EA, BM, and MI performed the experiments. FP, EA, MT, and PC analysed and interpreted the data. FP, MT, GS, and DM wrote the manuscript. DD, IM, and PC supervised the project. All authors contributed to the article and approved the submitted version.

## Conflict of Interest

DM was employed by company EMS—Elite Medical Service Ltd. The remaining authors declare that the research was conducted in the absence of any commercial or financial relationships that could be construed as a potential conflict of interest.

## Dedication

This article is dedicated to the memory of Massimo Marrelli, who died in Crotone, Italy, on October 27, 2018.
