# Supplementary material for: A Dedifferentiation Strategy to Enhance the Osteogenic Potential of Dental Derived Stem Cells
Source: Front Cell Dev Biol. 2021 May 28;9:668558. doi: 10.3389/fcell.2021.668558 (PMC8192975; doi:10.3389/fcell.2021.668558)
Supplement: Supplementary file 1 [file Data_Sheet_1.PDF]

Supplementary Material

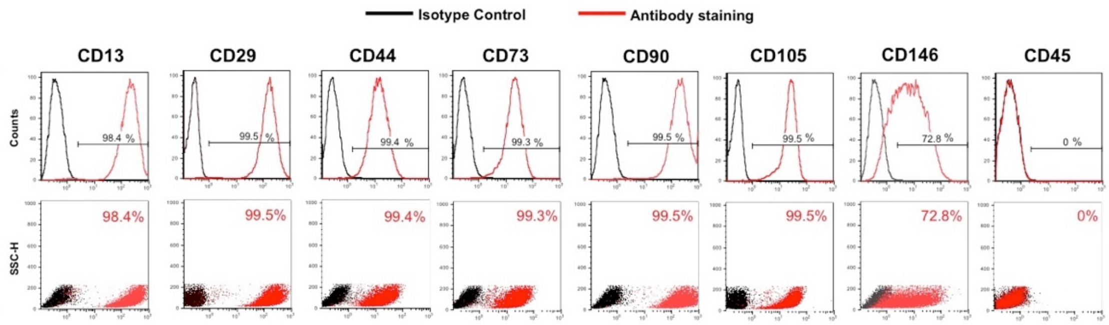

Supplementary Figure 1: Immunophenotype analysis of CD13, CD29, CD44, CD73, CD90, CD105, CD146 and CD45 expression in DFPCs at passage 2 by flow cytometry.

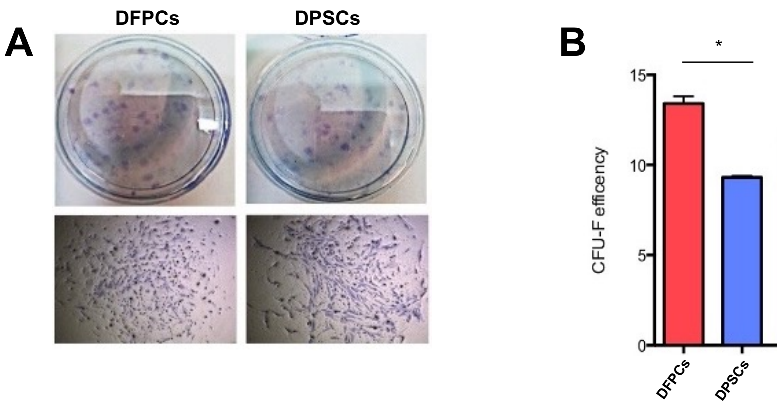

Supplementary Figure 2. Characterization of dental stem cells (DSCs): A) CFU-F (fibroblast colony-forming units). B) Quantification of CFU-F in DSCs.

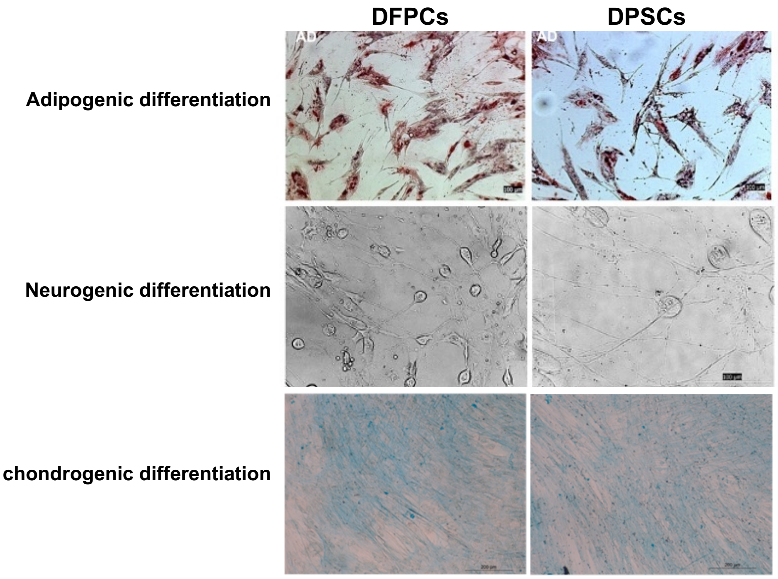

Supplementary Figure 3: Multilineage differentiation potential of DFPCs and DPSCs.

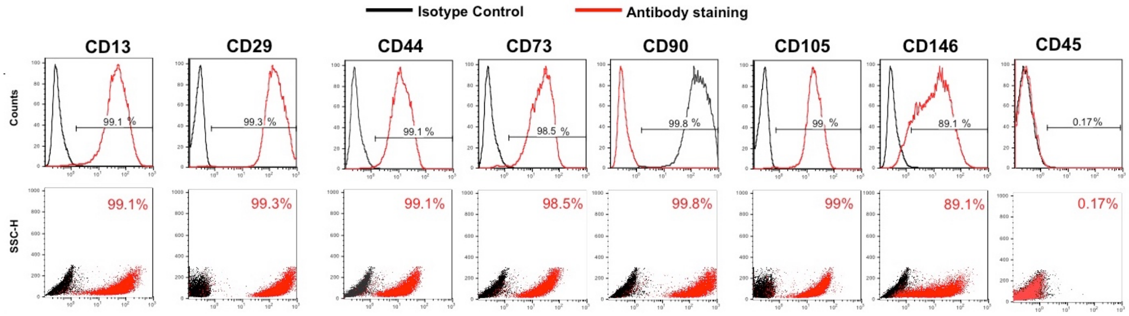

Supplementary Figure 4: Immunophenotype analysis of CD13, CD29, CD44, CD73, CD90, CD105, CD146 and CD45 expression in DPSCs at passage 2 by flow cytometry.

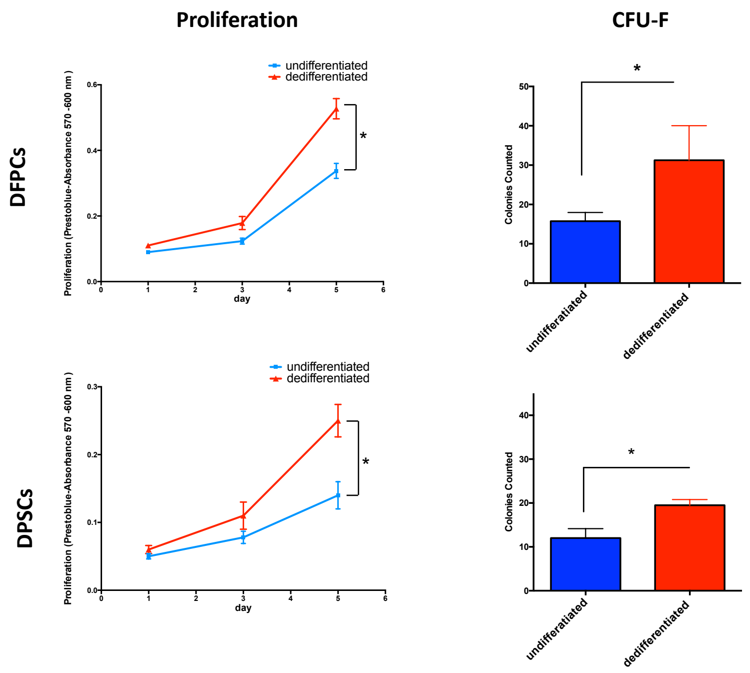

Supplementary Figure 5: Cell proliferation assay and CFU-F assay for Dediff-DFPCs and Dediff-DPSCs. Values were indicated statistically significant when *P<0.05.
